# Factors associated with physical and sexual violence among school-going adolescents in Nepal: Findings from Global School-based Student Health Survey

**DOI:** 10.1371/journal.pone.0248566

**Published:** 2021-03-18

**Authors:** Achyut Raj Pandey, Tamanna Neupane, Binaya Chalise, Niraj Shrestha, Sabina Chaudhary, Raja Ram Dhungana, Bihungum Bista

**Affiliations:** 1 Nepal Health Sector Programme 3, Monitoring, Evaluation and Operational Research, Abt Associates, Lalitpur, Nepal; 2 Nepal Health Research Council, Kathmandu, Nepal; 3 Graduate School for International Development and Cooperation, Hiroshima University, Hiroshima, Japan; 4 Unique College of Medical Science & Hospital, Saptari, Nepal; 5 Institute for Health and Sports, Victoria University, Victoria, Australia; National University of Singapore, SINGAPORE

## Abstract

**Background:**

Globally violence is a matter of public health concern with severe physical and mental health implications and social consequences. Evidence suggest that adolescents have an elevated risk of exposure to physical and sexual violence. However, there is a lack of nationally representative research on violence and its associated factors in Nepal to inform interventions. This paper attempts to find the factors associated with various forms of physical and sexual violence among school-going adolescents in Nepal.

**Methods:**

We analysed the cross-sectional data from the Global School-based Student Health Survey (GSHS) 2015. The GSHS survey applied a two-stage cluster sampling process to select a representative sample of 7 to 11 grade students from 74 schools across the country. We applied logistic regression analysis to identify the factors associated with physical and sexual violence.

**Results:**

Out of the total 6,529 participants, 45.24% of them faced a physical attack, 39.25% were involved in a physical fight, and 11.65% were victims of sexual violence in the survey administered between 7 August 2015 to 14 March 2016. In a multiple regression analysis, the age of participants, parental supervision, feeling unsafe at school, and the number of close friends were found to be associated with a physical attack. Participants who were bullied, had multiple sex partners, and had received corporal punishment in school had a higher engagement in a physical fight. Likewise, school grade, having parents who understand the problems, having multiple sex partners, and corporal punishment at school were associated with instances of sexual violence.

**Conclusion:**

The study identified multiple factors associated with experiences of physical attacks, involvement in a physical fight, and sexual violence among school-going adolescents. This study results can have important implications for school administration, parents, and policymakers alike to plan appropriate anti-violence strategies and interventions. Since various forms of violence share some common risk factors, a comprehensive strategy could be worth considering to prevent such acts of violence.

## Introduction

Adolescence is a period of transition. The changeover period exposes adolescents to vulnerable conditions and situations of violence, both as victims and aggressors [1,2]. With widening social interaction, adolescents start spending more time away from home, making them vulnerable to physical and sexual violence such as a physical attack, physical fight, and sexual harassment [2]. Furthermore, people in this age group are too young to unveil their experience of violence, which amplifies the problem and its consequences [3].

Recent evidence suggests that adolescents are disproportionately affected by physical and sexual violence in all settings across the world [4–8]. A study by Han et al. that analysed the data from 68 countries reported that 35.6% of school adolescents had faced a physical attack, and 36.4% were involved in physical fighting [9]. The prevalence of physical attack and physical fight were 39.7% and 28.1%, respectively, in the South-East Asian Region [9]. Evidence also suggests that globally approximately 9% of girls and 3% of boys are sexual violence victims [10]. An earlier study in Nepal had revealed that the prevalence of physical violence was 11% among women in the age group 15–19 years. In the same study, the proportion of adolescents women facing sexual violence in their lifetime was 3% [11]. Likewise, a study conducted among female students in a municipality of Kathmandu district reported that approximately 76% had faced some form of sexual harassment in their lifetime [12].

Violence is a deplorable act. However, evidence show that it is socially acceptable in some context. Approximately a third of the male adolescents in the rural settings (29%) and close to a quarter of the male adolescents in the urban settings (24%) justified wife-beating in Nepal [13]. Previous studies have revealed that factors such as being a male, smoking, consuming alcohol and drugs and bullying victimisation increase the odds of being involved in a physical fight, whereas parental supervision was found to have a protective effect [14]. Meanwhile, in a study among Kuwaiti adolescents, increased involvement in a physical fight was associated with food deprivation and truancy [15]. In a study, alcohol consumption and having friends who consume alcohol were associated with an increased risk of sexual violence [16]. Likewise, Ohene et al. had revealed the association of previous engagement in sexual activity and bullying victimisation with sexual violence among high school students [1].

Violent behaviour, primarily physical and sexual violence, has been a significant public health problem worldwide because of its severe personal and social consequences [2,8,17]. Violence amongst adolescents affects the victims’ lives in multiple ways and also have serious consewuences on family, community, and the nation [18–22]. Adolescents who experience physical and sexual violence have been found to have low social adjustment capacity compared to those not facing such violence [23]. Violence can affect the body systems, including the gastrointestinal, nervous, reproductive, cardiovascular, musculoskeletal, genitourinary, immune and endocrine systems [18]. It also contributes to the adoption of high-risk behaviour such as smoking, tobacco use, unhealthy diet and an eating disorder, harmful use of alcohol, and drug use that are considered a risk of non-communicable diseases [18]. In one of the previous studies, the adolescents who faced physical attacks and were involved in physical fights had almost two-fold higher odds of having serious injuries [24]. Similarly, those who had faced physical attacks had 1.38 times higher odds of having suicidal ideation and 2.42 times higher odds of suicidal attempts [25]. Apart from health outcomes, violence and its consequences also incur huge health care expenditure worldwide with substantial direct and indirect costs on the victims [5].

With increasing recognition of the burden of violence and its consequences, violence among adolescents has received global attention in recent years [19]. Given the global priority, it is necessary to estimate the prevalence of violence among adolescents to inform policy, drive actions against violence and monitor progress against the targets. Nepal lacks evidence from a representative nationwide study that shows the burden of violence and its associated factors. Having such evidence in the Nepalese context could help local, provincial, and federal governments formulate appropriate policy interventions to reduce the burden of violence and mitigate its impact. In this context, this study aimed to identify the factors associated with physical and sexual violence among school-going adolescents in Nepal.

## Methods

This study is a further analysis of the Global School-based Student Health Survey (GSHS) 2015. The GSHS survey applied a two-stage cluster sampling process to select a representative sample from the study population of 7 to 11 grade students. Firstly, the survey selected 74 schools based on probability proportional to school enrolment size from among the total schools that teach 7 to 11 grade students. Secondly, from 74 schools, an intact classroom was selected randomly, and the entire class was included in the study based on their eligibility criteria. Among 8,670 students approached for participation in the study, 6,531 participants completed the survey questionnaire. After the data cleaning procedure, 6,529 completed questionnaires were usable for data analyses.

Data were collected through the self-administration of a standardised questionnaire. The GSHS questionnaire used for this study contained 58 core questions, 33 expanded questions addressing all the GSHS core modules covering the students' demographics, dietary behaviours, hygiene, violence and unintentional injury, tobacco use, mental health, alcohol and drug use, sexual behaviours, and physical activity. The methodology has been detailed out in the full report of the study [26].

We classified participants as encountering physical attack if they had been attacked physically at least once in the past 12 months before the survey. Similarly, physical fighting refers to being involved in a physical fight at least once in the last 12 months. Participants were considered to have encountered sexual violence if they had been forced for sex when they did not want sexual intercourse ever in their lifetime. The definitions of outcome variables and selection of independent variables for analysis are based on previous publications from GSHS [8,27]. The definition of each study variable in our study is available in the supplementary file (S1 Table).

We used Stata 15 version to edit, process and analyse the data. We applied a weighting factor to each student's record to adjust for the non-response and for the varying probabilities of the participant's selection based on the sampling strategy. The weighing factor took into consideration the selection probability of school, the selection probability of classroom within the school, school level non-response and student-level non-response. Details of the calculation of the weighting factor are presented in a full report of the study [26].

We developed three multiple regression models: Model 1 (physical attack), Model 2 (physical fight) and Model 3 (sexual violence) and included all the independent variables in the models. Descriptive analysis and inferential analysis were performed using complex sample analysis considering the primary sample unit, strata, and each participant's selection probability. We performed separate regression analysis after imputation of missing data, and the results have been presented in a supplementary file (S2 Table).

The Ethical Review Board of Nepal Health Research Council ethically cleared the research. The student's participation in the study was voluntary, and the privacy and confidentiality of the research participants were ensured. After selecting the classrooms, all the students were provided with an information paper with detailed survey information to obtain voluntary written consent from parents or guardians. The following day, the survey question was administered among students having written informed consent. The students recorded their response on a computer scannable answer sheet during class hours.

## Results

### Characteristics of the participants

As shown in Table 1, the proportion of male and female participants in the study was 48.67% (95% CI = 46.55, 50.81) and 51.33% (95% CI = 49.19, 53.45) respectively. The proportion of participants with age 12 years or under was 11.99% (95% CI = 9.44, 15.12) and 16 years and above was 23.79% (95% CI = 21.10, 26.70). The proportion of participants ranged from 20.96% (95% CI = 16.36, 26.44) in grade ten or above to 27.70% (95% CI = 22.14, 34.04) in grade seven. Similarly, 4.68% (95% CI = 2.62, 8.23) had faced food insecurity, 50.86% (CI = 47.23, 54.47) had been bullied, 6.55% (95% CI = 5.45, 7.86) had felt lonely and 4.59% (CI = 3.73, 5.64) had anxiety. Almost two thirds 65.21% (95% CI = 61.20, 69.02) had at least three close friends. The proportion of participants with current cigarette use was 6.35% (95% CI = 4.87, 8.25), current alcohol use was 5.49% (95% CI = 4.20, 7.14) and current marijuana use was 3.23% (95% CI = 2.33, 4.47) (Table 1).

**Table 1:** Characteristics of research participants.

Variables	Attribute	n	Percentage (95% CI)
**Sex**	Male	3016	48.67 (46.55, 50.81)
	Female	3406	51.33 (49.19, 53.45)
**Age**	12 or under	606	11.99 (9.44, 15.12)
	13 years	1174	19.56 (17.96, 21.28)
	14 years	1504	24.55 (22.11, 27.18)
	15 years	1445	20.10 (18.29, 22.05)
	16 or above	1752	23.79 (21.10, 26.70)
**Grade**	Grade seven	1407	27.70 (22.14, 34.04)
	Grade eight	1830	27.68 (22.69, 33.29)
	Grade nine	1496	23.66 (19.00, 29.06)
	Grade ten or above	1707	20.96 (16.36, 26.44)
**Food insecurity**	No	6206	95.32 (91.77, 97.38)
	Yes	272	4.68 (2.62, 8.23)
**Physically attacked**	No	3685	54.76 (50.45, 59)
	Yes	2737	45.24 (41, 49.55)
**Physical fighting**	No	4051	60.75 (57.59, 63.81)
	Yes	2456	39.25 (36.19, 42.41)
**Bullied**	No	3213	49.14 (45.53, 52.77)
	Yes	2988	50.86 (47.23, 54.47))
**Felt lonely**	No	5986	93.45 (92.14, 94.56)
	Yes	402	6.55 (5.45, 7.86)
**Anxiety**	No	6206	95.41 (94.36, 96.28)
	Yes	282	4.59 (3.73, 5.64)
**Close friends**	No close friend	268	4.58 (3.28, 6.35)
	One close friend	833	13.99 (12.14, 16.08)
	Two close friends	990	16.22 (14.53, 18.07)
	Three or more close friends	4326	65.21 (61.20, 69.02)
**Current cigarette use**	No	6025	93.64 (91.75, 95.13)
	Yes	356	6.36 (4.87, 8.249)
**Current alcohol use**	No	6085	94.51 (92.86, 95.80)
	Yes	300	5.49 (4.20, 7.14)
**Ever used drug**	No	5712	91.12 (88.89, 92.93)
	Yes	495	8.88 (7.07, 11.11)
**Ever used marijuana**	No	6057	95.89 (94.2, 97.11)
	Yes	227	4.11 (2.89, 5.80)
**Current marijuana use**	No	6215	96.77 (95.53, 97.67)
	Yes	181	3.23 (2.33, 4.47)
**Multiple sex partners**	No	6047	96.21 (94.16, 97.56)
	Yes	228	3.79 (2.44, 5.84)
**Truancy**	0-2times	5834	90.63 (88.15, 92.64)
	3 times or more	524	9.37 (7.36, 11.85)
**Parents understand problem**	No	2838	47.39 (42.65, 52.18)
	Yes	3513	52.61 (47.82, 57.35)
**Parental monitoring**	No	2998	50.62 (45.87, 55.36)
	Yes	3422	49.38 (44.64, 54.13)
**Felt unsafe at school**	No	3933	58.28 (52.49, 63.85)
	Yes	2513	41.72 (36.15, 47.51)
**Corporal punishment**	No	3935	59.19 (56.17, 62.14)
	Yes	2496	40.81 (37.86, 43.83)
**Sexual violence**	No	5719	88.35 (85.42, 90.76)
	Yes	656	11.65 (9.24, 14.58)

The prevalence of physical attack was found to be 50.29% (95% CI = 45.50, 55.07) in males, 40.12% (95% CI = 35.45, 44.98) in females and 45.24% (95% CI = 41.00, 49.55) in both sexes. The prevalence of physical attack ranged from 40.81% (95% CI = 35.07, 46.81) among adolescents of age 16 years or above to 55.02% (95% CI = 47.09, 62.70) among adolescents of age 12 years or under. Similarly, the prevalence of physical attack ranged from 36.42% (95% CI = 30.29, 43.02) among adolescents in grade ten or above to 52.56% (95% CI = 45.96, 59.07) in grade seven (Table 2).

**Table 2:** Prevalence of physical attack, physical fight, and sexual violence.

Variables	Physical attack	Physical fight	Sexual violence
	Prevalence (95% CI)	Prevalence (95% CI)	Prevalence (95% CI)
**Sex**			
Male	50.29 (45.5, 55.07)	43.68 (40.01, 47.42)	11.95 (8.93, 15.81)
Female	40.12 (35.45, 44.98)	34.85 (30.84, 39.09)	11.08 (8.52, 14.29)
**Age**			
12 years or under	55.02 (47.09, 62.70)	35.53 (28.09, 43.74)	15.61 (11.01, 21.67)
13 years	47.95 (40.93, 55.04)	38.90 (34.22, 43.8)	8.80 (6.057, 12.63)
14 years	45.85 (41.16, 50.62)	41.03 (37.52, 44.62)	12.52 (8.85, 17.42)
15 years	40.84 (35.77, 46.1)	39.65 (35.92, 43.5)	10.92 (8.22, 14.36)
16 or above	40.81 (35.07, 46.81)	39.07 (35.53, 42.73)	11.64 (8.57, 15.62)
**Grade**			
Grade seven	52.56 (45.96, 59.07)	41.51 (35.18, 48.13)	16.09 (11.49, 22.06)
Grade eight	47.03 (41.59, 52.53)	37.93 (33.71, 42.35)	10.96 (8.29, 14.35)
Grade nine	42.72 (36.51, 49.16)	39.01 (32.54, 45.89)	9.58 (7.15, 12.71)
Grade ten or above	36.42 (30.29, 43.02)	38.65 (34.7, 42.75)	8.17 (5.60, 11.76)
**Food insecurity**			
No	44.51 (40.4, 48.71)	39.05 (35.75, 42.45)	11.34 (8.88, 14.38)
Yes	59.38 (52.68, 65.74)	42.55 (29.15, 57.15)	17.75 (14.36, 21.73)
**Bullied**			
No	30.24 (26.95, 33.75)	23.94 (20.55, 27.7)	9.54 (7.66, 11.82)
Yes	59.19 (53.92, 64.26)	54.33 (50.94, 57.68)	13.23 (9.54, 18.07)
**Felt lonely**			
No	44.21 (40.06, 48.44)	38.02 (34.92, 41.22)	10.86 (8.525, 13.75)
Yes	59.75 (52.24, 66.83)	52.73 (44.99, 60.33)	20.85 (15.29, 27.77)
**Anxiety**			
No	44.38 (40.25, 48.59)	38.78 (35.88, 41.77)	10.86 (8.53, 13.75)
Yes	59.54 (50.26, 68.19)	48.46 (39.7, 57.32)	20.85 (15.29, 27.77)
**Close friends**			
No close friend	38.01 (29.34, 47.53)	48.40 (37.7, 59.28)	21.57 (14.79, 30.34)
One close friend	46.24 (40.61, 51.97)	40.88 (34.53, 47.55)	13.95 (10.33, 18.56)
Two close friends	45.90 (39.83, 52.08)	39.09 (34.37, 44.02)	15.7 (12.37, 19.72)
Three or more close friends	45.22 (40.78, 49.74)	38.18 (34.83, 41.65)	9.13 (6.81, 12.12)
**Current cigarette use**			
No	43.28 (39.29, 47.35)	37.12 (34.15, 40.18)	10.19 (8.27, 12.49)
Yes	70.16 (61.09, 77.88)	65.38 (55.86, 73.8)	30.42 (21.72, 40.78)
**Current alcohol use**			
No	43.72 (39.69, 47.83)	37.49 (34.59, 40.48)	10.44 (8.59, 12.64)
Yes	61.24 (48.65, 72.48)	61.46 (51.59, 70.46)	21.92 (13.85, 32.91)
**Ever used drug**			
No	43.1 (38.97, 47.33)	36.22 (33.09, 39.47)	9.982 (7.78, 12.72)
Yes	59.13 (52.06, 65.83)	58.82 (53.55, 63.90)	20.8 (15.1, 27.94)
**Current marijuana use**			
No	43.97 (39.92, 48.09)	37.83 (34.78, 40.98)	10.98 (8.73, 13.73)
Yes	70.55 (61.2, 78.45)	68.24 (58.67, 76.49)	32.35 (23.78, 42.31)
**Multiple sex partner**			
No	44.26 (40.18, 48.42)	37.58 (34.24, 41.03)	10.76 (8.66, 13.29)
Yes	55.8 (45.43, 65.68)	70.19 (57.91, 80.13)	26.7 (19.14, 35.91)
**Truancy**			
0–2 times	43.79 (39.7, 47.96)	37.89 (34.59, 41.31)	10.67 (8.54, 13.26)
3 times or more	56.49 (49.38, 63.34)	49.38 (40.5, 58.29)	19.77 (15.15, 25.39)
**Parents understand problem**			
No	49.37 (44.45, 54.31)	40.48 (37.6, 43.44)	15.26 (12.02, 19.18)
Yes	41.11 (37.33, 45.00)	37.46 (34.03, 41.02)	7.725 (6.45, 9.23)
**Parental supervision**			
No	51.31 (46.92, 55.68)	42.91 (39.35, 46.54)	15.04 (12.15, 18.46)
Yes	38.84 (35.27, 42.54)	35.08 (31.81, 38.51)	7.877 (6.41, 9.65)
**Felt unsafe in school**			
No	36.59 (33.04, 40.29)	34.88 (31.21, 38.74)	8.536 (6.62, 10.94)
Yes	57.50 (52.61, 62.24)	45.2 (42.24, 48.2)	15.55 (12.27, 19.52)
**Corporal punishment**			
No	38.3 (33.94, 42.86)	32.94 (29.6, 36.45)	7.50 (5.67, 9.86)
Yes	54.55 (49.74, 59.28)	48.31 (44.86, 51.79)	17.41 (13.78, 21.75)
**Prevalence**	**45.24 (41.00, 49.55)**	**39.25 (36.19, 42.41)**	**11.65 (9.24, 14.58)**

The prevalence of physical fight was 43.68% (95% CI = 40.01, 47.42) among males, 34.85% (95% CI = 30.84, 39.09) amongst female and 39.25% (95% CI = 36.19, 42.41) in both sexes. The prevalence of physical fight ranged from 35.53% (95% CI = 28.09, 43.74) among adolescents under 12 years of age to 41.03% (95% CI = 37.52, 44.62) among adolescents of age 14 years. Similarly, the prevalence of physical fight ranged from 37.93% (95% CI = 33.71, 42.35) among adolescents in grade eight to 41.51% (95% CI = 35.18, 48.13) among adolescents in grade seven (Table 2).

The overall prevalence of sexual violence was 11.65% (95% CI = 9.24, 14.58). Gender-wise disaggregation shows that 11.95% (95% CI = 8.93, 15.81) of males and 11.08% (95% CI = 8.52, 14.29) of females had faced sexual violence. Sexual violence ranged from 8.80% (95% CI = 6.057, 12.63) among adolescents of age 13 years to 15.61% (95% CI = 11.01, 21.67) among adolescents 12 years or below. Similarly, sexual violence ranged from 8.17% (95% CI = 5.60, 11.76) among adolescents of grade ten or above to 16.09% (95% CI = 11.49, 22.06) among adolescents in grade seven (Table 2).

In bivariate analysis, a statistically significant association was found between age and being physically attacked. Compared to adolescents of age 12 years or under, adolescents of age 14 years (OR = 0.69, 95% CI = 0.51, 0.94), 15 years (OR = 0.56, 95% CI = 0.40, 0.79), and 16 years or above (OR = 0.56, 95% CI = 0.40, 0.80) had lower odds of being physically attacked. Female adolescents were less likely to be physically attacked (OR = 0.66, 95% CI = 0.55, 0.79) and getting engaged in a physical fight (OR = 0.69, 95% CI = 0.56, 0.85) compared to males. Adolescents who had faced food insecurity had 1.82 times (OR = 1.82, 95% CI = 1.38, 2.41) and 1.69 times (OR = 1.69, 95% CI = 1.08, 2.64) higher odds of being physically attacked and being forced for sexual intercourse, respectively. Bullied participants had 3.35 times higher odds (OR = 3.35, 95% CI = 2.78, 4.02) of being physically attacked, 3.78 times higher odds (OR = 3.78, 95% CI = 3.06, 4.66) of being involved in a physical fight and 1.45 times higher odds (OR = 1.45 95% CI = 1.01, 2.07) of facing sexual violence. Adolescents who felt lonely had 1.87 times higher odds (OR = 1.87, 95% CI = 1.45, 2.42) of being physically attacked, 1.82 times higher odds (OR = 1.82, 95% CI = 1.34, 2.47) of being involved in a physical fight and 2.16 times higher odds (OR = 2.16, 95% CI = 1.46, 3.19) of facing sexual violence. Adolescents who had anxiety had 1.84 times higher odds (OR = 1.84, 95% CI = 1.32, 2.58) of being physically attacked, 1.48 times higher odds (OR = 1.48, 95% CI = 1.11, 1.98) of being involved in a physical fight and 2.83 times higher odds (OR = 2.83, 95% CI = 1.53, 5.23) of facing sexual violence. Similarly, adolescents involved in tobacco use, alcohol use, marijuana use had higher odds of being physically attacked, being involved in a physical fight and encountering sexual violence. The association of other variables with being physically attacked, being involved in a physical fight, and facing sexual violence are shown below (Table 3).

**Table 3:** Factors associated with physical attack, physical fight, and sexual violence in bivariate analysis.

Variables	Physical attack		Physical fight		Sexual Violence	
	OR (95% CI)	*p-*value	OR (95% CI)	p-value	OR (95% CI)	p value
**Sex**						
Male	Ref		Ref		Ref	
Female	0.66 (0.55, 0.79)	<0.01	0.69 (0.56, 0.85)	<0.01	0.92 (0.67, 1.27)	0.59
**Age**						
12 years or under	Ref		Ref		Ref	
13 years	0.75 (0.57, 1.00)	0.05	1.16 (0.83, 1.61)	0.38	0.52 (0.30, 0.92)	0.03
14 years	0.69 (0.51, 0.94)	0.02	1.26 (0.91, 1.76)	0.16	0.77 (0.45, 1.32)	0.34
15 years	0.56 (0.40, 0.79)	<0.01	1.19 (0.82, 1.73)	0.34	0.66 (0.41, 1.07)	0.09
16 and above	0.56 (0.40, 0.80)	<0.01	1.16 (0.86, 1.57)	0.31	0.71 (0.46, 1.10)	0.12
**Grade**						
Grade seven	Ref		Ref		Ref	
Grade eight	0.80 (0.59, 1.08)	0.15	0.86 (0.62, 1.21)	0.37	0.64 (0.42, 0.99)	0.05
Grade nine	0.67 (0.50, 0.91)	0.01	0.90 (0.62, 1.31)	0.58	0.55 (0.40, 0.76)	<0.01
Grade ten or above	0.52 (0.38, 0.70)	<0.01	0.89 (0.67, 1.18)	0.40	0.46 (0.28, 0.77)	<0.01
**Food insecurity**						
No	Ref		Ref		Ref	
Yes	1.82 (1.38, 2.41)	<0.01	1.16 (0.62, 2.17)	0.64	1.69 (1.08, 2.64)	0.02
**Bullied**						
No	Ref		Ref		Ref	
Yes	3.35 (2.78, 4.02)	<0.01	3.78 (3.06, 4.66)	<0.01	1.45 (1.01, 2.07)	0.04
**Felt lonely**						
No	Ref		Ref		Ref	
Yes	1.87 (1.45, 2.42)	<0.01	1.82 (1.34, 2.47)	<0.01	2.16 (1.46, 3.19)	<0.01
**Anxiety**						
No	Ref		Ref		Ref	
Yes	1.84 (1.32, 2.58)	<0.01	1.48 (1.11, 1.98)	0.01	2.83 (1.53, 5.23)	<0.01
**Close friends**						
No close friend	Ref		Ref		Ref	
One close friend	1.40 (0.93, 2.12)	0.11	0.74 (0.45, 1.21)	0.22	0.59 (0.33, 1.04)	0.07
Two close friends	1.38 (0.90, 2.13)	0.14	0.68 (0.44, 1.06)	0.09	0.68 (0.40, 1.14)	0.14
Three or more close friends	1.35 (0.93, 1.95)	0.11	0.66 (0.43, 1.02)	0.06	0.37 (0.20, 0.68)	<0.01
**Current cigarette use**						
No	Ref		Ref		Ref	
Yes	3.08 (2.10, 4.52)	<0.01	3.20 (2.13, 4.81)	<0.01	3.85 (2.78, 5.34)	<0.01
**Current alcohol use**						
No	Ref		Ref		Ref	
Yes	2.03 (1.25, 3.31)	0.01	2.66 (1.79, 3.94)	<0.01	2.41 (1.48, 3.91)	<0.01
**Ever used drug**						
No	Ref		Ref		Ref	
Yes	1.91 (1.45, 2.51)	<0.01	2.52 (2.08, 3.05)	<0.01	2.37 (1.59, 3.53)	<0.01
**Current marijuana use**						
No	Ref		Ref		Ref	
Yes	3.05 (2.02, 4.62)	<0.01	3.53 (2.31, 5.40)	<0.01	3.88 (2.68, 5.60)	<0.01
**Multiple sex partner**						
No	Ref		Ref		Ref	
Yes	1.59 (1.04, 2.44)	0.04	3.91 (2.20, 6.96)	<0.01	3.02 (2.08, 4.39)	<0.01
**Truancy**						
0–2 times	Ref		Ref		Ref	
3 times or more	1.67 (1.27, 2.18)	<0.01	1.6 (1.07, 2.38)	0.02	2.06 (1.51, 2.81)	<0.01
**Parents understand problem**						
No	Ref		Ref		Ref	
Yes	0.72 (0.61, 0.84)	<0.01	0.88 (0.78, 0.99)	0.03	0.46 (0.37, 0.58)	<0.01
**Parental supervision**						
No	Ref		Ref		Ref	
Yes	0.6 (0.53, 0.69)	<0.01	0.72 (0.61, 0.85)	<0.01	0.48 (0.40, 0.59)	<0.01
**Felt unsafe in school**						
No	Ref		Ref		Ref	
Yes	2.34 (1.98, 2.78)	<0.01	1.54 (1.32, 1.80)	<0.01	1.97 (1.51, 2.58)	<0.01
**Corporal punishment**						
No	Ref		Ref		Ref	
Yes	1.93 (1.62, 2.3)	<0.01	1.9 (1.58, 2.29)	<0.01	2.60 (2.07, 3.26)	<0.01

Sex of the participants was found to have a statistically significant association with being physically attacked with females (AOR = 0.79, 95% CI = 0.68, 0.92) having lower odds compared to males. The participants of the age group 15 years (AOR = 0.64, 95% CI = 0.43, 0.95) and 16 years or above (AOR = 0.62, 95% CI = 0.39, 0.98) had lower odds of being physically attacked compared to participants of age 12 years or under. Those who were bullied had 2.65 times higher odds (AOR = 2.65, 95% CI = 2.15, 3.26) of being physically attacked compared to their counterparts who were not bullied. Participants who had three or more friends had 1.65 times higher odds (AOR = 1.65, 95% CI = 1.07, 2.53) of being physically attacked than those not having any close friends. Adolescents with parental supervision had lower odds (AOR = 0.83, 95% CI = 0.70, 0.99) of being physically attacked than those who did not. Those who felt unsafe in school had almost 1.91 times higher odds (AOR = 1.91, 95% CI = 1.55, 2.34) of being physically attacked compared to their other counterparts. Similarly, those who had received corporal punishment like being hit, slapped, or were physically hurt by the teacher were found to have 1.40 times higher odds (AOR = 1.40, 95% CI = 1.17, 1.67) of being physically attacked compared to other counterparts (Table 4).

**Table 4:** Factors associated with physical attack, physical fight, and sexual violence in multivariate analysis.

Variables	Physical attack (n = 4943)		Physical fight (n = 4993)		Sexual Violence (n = 4959)	
	AOR (95% CI)	*p-*value	AOR (95% CI)	p-value	AOR (95% CI)	p value
**Sex**						
Male	Ref		Ref		Ref	
Female	0.79 (0.68, 0.92)	<0.01	0.80 (0.64, 1.00)	0.05	1.19 (0.86, 1.65)	0.28
**Age**						
12 years or under	Ref		Ref		Ref	
13 years	0.77 (0.53, 1.1)	0.15	1.28 (0.86, 1.92)	0.22	0.66 (0.37, 1.19)	0.16
14 years	0.72 (0.5, 1.04)	0.08	1.37 (0.84, 2.25)	0.20	1.21 (0.61, 2.41)	0.57
15 years	0.64 (0.43, 0.95)	0.03	1.21 (0.73, 2.02)	0.45	1.48 (0.82, 2.66)	0.18
16 and above	0.62 (0.39, 0.98)	0.04	0.98 (0.61, 1.6)	0.95	1.34 (0.77, 2.35)	0.30
**Grade**						
Grade seven	Ref		Ref		Ref	
Grade eight	0.95 (0.7, 1.29)	0.75	0.89 (0.65, 1.22)	0.47	0.62 (0.35, 1.09)	0.10
Grade nine	0.88 (0.64, 1.22)	0.43	1.11 (0.78, 1.58)	0.56	0.51 (0.32, 0.81)	0.01
Grade ten or above	0.71 (0.49, 1.01)	0.05	1.09 (0.82, 1.46)	0.55	0.34 (0.20, 0.60)	<0.01
**Food Insecurity**						
No	Ref		Ref		Ref	
Yes	1.22 (0.87, 1.7)	0.24	0.67 (0.38, 1.17)	0.15	1.02 (0.45, 2.32)	0.96
**Bullied**						
No	Ref		Ref		Ref	
Yes	2.65 (2.15, 3.26)	<0.01	3.39 (2.75, 4.18)	<0.001	1.10 (0.75, 1.61)	0.62
**Felt lonely**						
No	Ref		Ref		Ref	
Yes	1.25 (0.86, 1.80)	0.24	1.22 (0.78, 1.93)	0.37	1.20 (0.68, 2.11)	0.52
**Anxiety**						
No	Ref		Ref		Ref	
Yes	1.06 (0.72, 1.56)	0.75	0.96 (0.61, 1.51)	0.86	2.02 (0.95, 4.3)	0.07
**Close friends**						
No close friend	Ref		Ref		Ref	
One close friend	1.58 (0.95, 2.63)	0.08	0.87 (0.51, 1.48)	0.60	1.03 (0.48, 2.21)	0.94
Two close friends	1.46 (0.9, 2.35)	0.12	0.81 (0.49, 1.34)	0.40	1.23 (0.55, 2.79)	0.61
Three or more close friends	1.65 (1.07, 2.53)	0.02	0.76 (0.47, 1.23)	0.25	0.71 (0.34, 1.5)	0.36
**Current cigarette use**						
No	Ref		Ref		Ref	
Yes	1.49 (0.94, 2.36)	0.09	1.25 (0.7, 2.23)	0.44	1.75 (0.8, 3.81)	0.16
**Current alcohol use**						
No	Ref		Ref		Ref	
Yes	1.04 (0.56, 1.95)	0.89	1.18 (0.76, 1.82)	0.44	1.24 (0.58, 2.64)	0.57
**Ever used drug**						
No	Ref		Ref		Ref	
Yes	0.75 (0.49, 1.15)	0.18	1.17 (0.8, 1.71)	0.41	0.96 (0.37, 2.5)	0.93
**Current marijuana use**						
No	Ref		Ref		Ref	
Yes	1.51 (0.81, 2.83)	0.19	2.28 (0.93, 5.55)	0.07	1.08 (0.33, 3.53)	0.90
**Multiple sex partner**						
No	Ref		Ref		Ref	
Yes	0.84 (0.52, 1.38)	0.49	2.33 (1.06, 5.14)	0.04	2.02 (1.15, 3.54)	0.02
**Truancy**						
0–2 times	Ref		Ref		Ref	
3 times or more	1.38 (0.99, 1.91)	0.06	1.31 (0.83, 2.07)	0.24	1.28 (0.8, 2.06)	0.29
**Parents understand problem**						
No	Ref		Ref		Ref	
Yes	0.91 (0.74, 1.13)	0.38	1.07 (0.92, 1.25)	0.36	0.68 (0.53, 0.87)	<0.01
**Parental supervision**						
No	Ref		Ref		Ref	
Yes	0.83 (0.7, 0.99)	0.04	0.91 (0.73, 1.14)	0.40	0.77 (0.59, 1.00)	0.05
**Felt unsafe in school**						
No	Ref		Ref		Ref	
Yes	1.91 (1.55, 2.34)	<0.01	1.09 (0.89, 1.34)	0.39	1.37 (0.98, 1.9)	0.06
**Corporal punishment**						
No	Ref		Ref		Ref	
Yes	1.40 (1.17, 1.67)	<0.01	1.56 (1.25, 1.94)	<0.001	2.38 (1.73, 3.27)	<0.01

Those who were bullied had almost 3.39 times higher odds (AOR = 3.39, 95% CI = 2.75, 4.18) of being involved in a physical fight compared to adolescents who were not bullied. Similarly, those with multiple sex partners had around 2.33 times higher odds (AOR = 2.33, 95% CI = 1.06, 5.14) of being involved in a physical fight compared to those who did not have multiple sex partners. Similarly, compared to their other counterparts, adolescents who had received corporal punishment from teachers in school had 1.56 times higher odds (AOR = 1.56, 95% CI = 1.25, 1.94) of being involved in a physical fight (Table 4).

Adolescents in grade nine (AOR = 0.51, 95% CI = 0.32, 0.81) and grade ten or above (AOR = 0.34, 95% CI = 0.20, 0.60) had lower odds of having sexual violence compared to adolescents in grade seven. Adolescents who had multiple sex partners had 2.02 times higher odds (AOR = 2.02, 95% CI = 1.15, 3.54) of facing sexual violence than their counterparts. Adolescents whose parents understand their problems had lower odds (AOR = 0.68, 95% CI = 0.53, 0.87) of facing sexual violence compared to their other counterparts. Corporal punishment in school was also found to have a statistically significant association with sexual violence. Those who faced corporal punishment had 2.38 times higher odds (AOR = 2.38, 95% CI = 1.73, 3.27) of facing sexual violence (Table 4).

## Discussion

Our study showed that physical fight, physical attack, and sexual violence among adolescents are associated with several socio-demographic, behavioural and other factors. For example, male sex, having three and more close friends, not having parental supervision, unsafe feeling in school, and corporal punishment were found to have higher odds of experiencing a physical attack. The results showed that being a victim of bullying and corporal punishment are the common factor statistically associated with a physical attack, physical fight, and sexual violence.

As per a multi-country study, the prevalence of physical fights among school-going adolescents was 21.2% in Bangladesh, 25.1% in Indonesia, 28.4% in Thailand, 32.1% in the Maldives, and 33.6% in Timor-Leste [9]. Likewise, our study revealed that 39.5% of school-going adolescents were involved in a physical fight in Nepal. Similarly, the proportion of school-going adolescents reporting physical attacks differed across countries. The prevalence was 9.8% in Thailand, 31.5% in the Maldives, 34.3% in Indonesia, 41.1% in Timor-Leste, and 62.5% in Bangladesh [9]. In Nepal, we found that 45.24% of school-going adolescents were physically attacked. An earlier study suggests that poverty level, cultural factors, substance use, and educational attainment could have led to intercountry variation in violence prevalence [9]. In the future, multi-country studies examining factors responsible for variations in prevalence, despite complexities in comparing the socio-economic, cultural, and behavioural indicators across countries, could be helpful.

Studies from Oman [28], Venezuela [29], Namibia [27] and Egypt [30] report that females, compared to males, have lower odds of being physically attacked. We did explore the gender differences associated with different forms of violence in our study through bivariate and multivariate analysis. Both the analysis show that females are less likely to be physically attacked than males. Our study also shows that the likelihood of females engaging in a physical fight was lesser than males in bivariate analysis. However, multivariate analysis shows that the association between participants' sex and engagement in a physical fight is borderline. The social acceptance of male engagement in physical violence can be one factor contributing to gender differences. A study found that boys were more likely to engage in physical aggression like physical fighting while the girls are more likely to show verbal aggression and engage in indirect forms of violence [27], indicating that the nature of violence may vary with the sex of the participants. Hence, similar studies in future can look to examine if the forms of violence differ based on gender.

Parental supervision was found to reduce the odds of being physically attacked. Previous studies indicate that having a close parent-child relationship may improve youth's ability to select pro-social friends [31] reduce the risk of substance use, aggression, and risky sexual behaviour [32], which could ultimately reduce the risk of involvement in different forms of violence. Furthermore, the extent to which parents exercise control over children's different behaviours might create a difference in the association.

We found that bullied children were almost three times more likely to be involved in a physical fight and be physically attacked. Studies from Venezuela [29], the USA [33] and Egypt [30] also report an association between being bullied and being involved in a physical fight. Furthermore, a US study reports that bullied children are more likely to carry weapons or act as perpetrators than those not bullied [34]. Likewise, we also found that school-going adolescents who receive corporal punishment from teachers in a school are also more likely to be physically attacked, get involved in a physical fight, and become victims of sexual violence. We observed that adolescents are often victims of multiple forms of violence. However, because of the cross-sectional nature of data, cause and effect relationship cannot be established. Nevertheless, the co-occurrence of multiple forms of violence may also be related to harmful peer behaviour among school students, as indicated in the earlier studies [35].

In our study, the prevalence of sexual violence was similar in males and females; 11.95% of the male participants and 11.08% of the female participants had faced sexual violence which is slightly higher than the prevalence of sexual violence in Bhutan (7.1%, both sexes) [36]. Although we did not find any statistically significant association between participants' sex and sexual violence, earlier studies have reported that females are more likely to suffer sexual violence than males [8,37].

The study found that adolescents in grade eight or nine seem to be less likely to be forced for sex than adolescents in grade seven. One of the factors might be that, as the education level increases, adolescents tend to be more aware of others' sexual intentions and better understand how to avoid such circumstances where adolescents are forced into sex. Furthermore, the perpetrator might have been reluctant in victimising adolescents in higher grades as they might be aware of legal procedures and seek legal and other solutions if forced for sex. Further studies on the topic could be useful in elucidating how these variables are associated.

We found that those with multiple sex partners had almost two-fold higher odds of being engaged in a physical fight and almost two-fold higher odds of encountering sexual violence. A study by Stockman et al. found that having multiple sex partners was associated with sexual violence [21]. Similarly, those who were intoxicated with alcohol or drug during sexual violence (coerced sex) had a higher risk of having multiple sex partners [21]. Another multi-country study from Africa found that those who had faced sexual violence had a higher risk of having multiple sex partners [8]. Nonetheless, we cannot comment on whether sexual violence leads to multiple sex partners or having multiple sex partners increases the risk of sexual violence because it is a cross-sectional study.

The study has some limitations. It is limited to school-going adolescents and does not cover any children out of school who may be equally or more likely to face physical and sexual violence. The study suffers from recall bias because it is a self-reported survey, and participants might have had problems recalling their experience of violence vividly and understanding the questions in some cases. Further, the study does not provide a cause and effect relationship because it is a cross-sectional study. Nevertheless, this is the first nationwide study among adolescents exploring violence and its associated factors.

Overall, the study suggests that school-going adolescents are exposed to multiple forms of violence. Therefore, it is essential to draft a comprehensive strategy that prevents school-going adolescents from all forms of violence and prioritises creating an environment free from violence. For example, as part of their curriculum, students could be taught to deal with violence in different settings. Furthermore, making students aware of when they should seek legal support and from where they can seek such support could be useful. Schools can be encouraged to create a violence-free environment through appropriate incentives from the local governments. Nonetheless, from a research perspective focus should be to understand how physical and sexual violence occur in a different setting with a broader set of social and cultural variables covering adolescents both in and out of school.

## Conclusion

Sex, age, having three or more close friends, parental supervision, feeling unsafe at school, and receiving corporal punishment were significantly associated with being physically attacked. Similarly, having multiple sex partners was associated with involvement in a physical fight and encountering sexual violence. Among other factors associated with sexual violence, the study participant's school grade was one of them. The school grade was significantly associated with sexual violence. Students from higher grades were found to have lower odds of facing sexual violence. Being bullied, receiving corporal punishment in school were associated with all forms of violence: physical attack, being involved in a physical fight, physical attack, and sexual violence. Since different forms of violence share some common risk factors, a comprehensive strategy could prevent violence.

## Supporting information

S1 Table Operational definition of the variables in the study. (DOC)

S2 Table Results of multiple regression analysis after imputation of missing data. (DOC)

## References

[1] Ohene SA, Johnson K, Atunah-Jay S, Owusu A, Borowsky IW. Sexual and physical violence victimization among senior high school students in Ghana: Risk and protective factors. Social Science & Medicine. 2015;146:266–75. 10.1016/j.socscimed.2015.10.019 26603310

[2] United Nations Children’s Fund. A Familiar Face. Violence in the lives of children and adolescents, New York. UNICEF; 2017.

[3] United Nations Children’s Fund. Hidden in Plain Sight: a statistical analysis of violence against children. 2015.

[4] World Health Organization. Management of Substance Abuse Unit. Global status report on alcohol and health, 2014: World Health Organization; 2014.

[5] World Health Organization. World report on violence and health. 2002.

[6] Rosenberg ML, Butchart A, Mercy J, Narasimhan V, Waters H, Marshall MS. Interpersonal violence. 2006.

[7] Black MC, Basile KC, Breiding MJ, Smith SG, Walters ML, Merrick MT, et al. The national intimate partner and sexual violence survey: 2010 summary report. Atlanta: National Center for Injury Prevention and Control, Centers for Disease Control and Prevention, 2011.

[8] Brown DW, Riley L, Butchart A, Meddings DR, Kann L, Harvey AP. Exposure to physical and sexual violence and adverse health behaviours in African children: results from the Global School-based Student Health Survey. Bulletin of the World Health Organization. 2009;87:447–55. 10.2471/blt.07.047423 19565123 PMC2686210

[9] Han L, You D, Gao X, Duan S, Hu G, Wang H, et al. Unintentional injuries and violence among adolescents aged 12–15 years in 68 low-income and middle-income countries: a secondary analysis of data from the Global School-Based Student Health Survey. The Lancet Child & Adolescent Health. 2019;3(9):616–26. 10.1016/S2352-4642(19)30195-6 31278043

[10] Barth J, Bermetz L, Heim E, Trelle S, Tonia T. The current prevalence of child sexual abuse worldwide: a systematic review and meta-analysis. International journal of public health. 2013;58(3):469–83. 10.1007/s00038-012-0426-1 23178922

[11] Ministry of Health—MOH/Nepal, New ERA/Nepal, ICF. Nepal Demographic and Health Survey 2016. Kathmandu, Nepal: MOH/Nepal, New ERA, and ICF, 2017.

[12] Thapalia R, Dhungana RR, Adhikari SK, Pandey AR. Understanding, experience and response to sexual harassment among the female students: a mixed method study. Journal of Nepal Health Research Council. 2019;17(4):424–30. 10.33314/jnhrc.v17i4.2313 32001843

[13] Dalal K, Lee MS, Gifford M. Male adolescents' attitudes toward wife beating: a multi-country study in South Asia. Journal of Adolescent Health. 2012;50(5):437–42. 10.1016/j.jadohealth.2011.09.012 22525105

[14] Rudatsikira E, Muula AS, Siziya SB. Prevalence and correlates of physical fighting among school-going adolescents in Santiago, Chile. razilian Journal of Psychiatry. 2008;30(3):197–202. 10.1590/s1516-44462008000300004 18833418

[15] Shaikh MA, Abio AP, Adedimeji AA, Lowery Wilson M. Involvement in physical fights among school attending adolescents: a nationally representative sample from Kuwait. Behavioral Sciences. 2020;10(1):29. 10.3390/bs10010029 31936281 PMC7016681

[16] Adinew YM, Hagos MA. Sexual violence against female university students in Ethiopia. BMC international health human rights. 2017;17(1):1–7. 10.1186/s12914-016-0109-8 28738807 PMC5525286

[17] Breiding MJ, Smith SG, Basile KC, Walters ML, Chen J, Merrick MT. Prevalence and characteristics of sexual violence, stalking, and intimate partner violence victimization—national intimate partner and sexual violence survey, United States, 2011. Morbidity and mortality weekly report Surveillance summaries. 2014;63(8):1–18. Epub 2014/09/05. 25188037 PMC4692457

[18] Sen P, Jewkes R, Garcia-Moreno C. Sexual violence. 2002.

[19] Hillis S, Mercy J, Amobi A, Kress H. Global prevalence of past-year violence against children: a systematic review and minimum estimates. Pediatrics. 2016;137(3):e20154079. 10.1542/peds.2015-4079 26810785 PMC6496958

[20] Hughes K, Bellis MA, Hardcastle KA, Sethi D, Butchart A, Mikton C, et al. The effect of multiple adverse childhood experiences on health: a systematic review and meta-analysis. The Lancet Public health. 2017;2(8):e356–e66. Epub 2017/12/19. 10.1016/S2468-2667(17)30118-4 . 29253477

[21] Stockman JK, Campbell JC, Celentano DD. Sexual violence and HIV risk behaviors among a nationally representative sample of heterosexual American women: The importance of sexual coercion. Journal of acquired immune deficiency syndromes (1999). 2010;53(1):136. 10.1097/QAI.0b013e3181b3a8cc 19734802 PMC2799543

[22] Lundgren R, Amin A. Addressing intimate partner violence and sexual violence among adolescents: emerging evidence of effectiveness. The Journal of adolescent health: official publication of the Society for Adolescent Medicine. 2015;56(1):S42–50. Epub 2014/12/23. 10.1016/j.jadohealth.2014.08.012 . 25528978

[23] Deb S, Walsh K. Impact of physical, psychological, and sexual violence on social adjustment of school children in India. School Psychology International. 2012;33(4):391–415. 10.1177/0143034311425225

[24] Pandey AR, Neupane T, Chalise B, Chaudhary S, Shrestha N, Bista B. Serious Injury and its Correlates among School Going Adolescents in Nepal: A cross-sectional study. Journal of Nepal Health Research Council. 2020;18(3):506–12. Epub 2020/11/20. 10.33314/jnhrc.v18i3.2882 . 33210649

[25] Pandey AR, Bista B, Dhungana RR, Aryal KK, Chalise B, Dhimal M. Factors associated with suicidal ideation and suicidal attempts among adolescent students in Nepal: Findings from Global School-based Students Health Survey. PloS one. 2019;14(4):e0210383. Epub 2019/04/20. 10.1371/journal.pone.0210383 31002715 PMC6474648

[26] Aryal KK, Bista B, Khadka BB, Dhimal M, Pandey AR, Mehta R, et al. Global School Based Student Health Survey Nepal, 2015. Kathmandu: Nepal Health Research Council, 2016.

[27] Rudatsikira E, Siziya S, Kazembe LN, Muula AS. Prevalence and associated factors of physical fighting among school-going adolescents in Namibia. Annals of General Psychiatry. 2007;6(1):18. 10.1186/1744-859X-6-18 17650328 PMC1947983

[28] Peyton RP, Ranasinghe S, Jacobsen KH. Injuries, violence, and bullying among middle school students in Oman. Oman medical journal. 2017;32(2):98. 10.5001/omj.2017.19 28439379 PMC5397083

[29] Muula AS, Herring P, Siziya S, Rudatsikira E. Bullying victimization and physical fighting among Venezuelan adolescents in Barinas: results from the Global School-Based Health Survey 2003. Italian Journal of Pediatrics. 2009;35(1):38. 10.1186/1824-7288-35-38 19939261 PMC2789090

[30] Celedonia KL, Wilson ML, El Gammal HA, Hagras AM. Physical fighting among Egyptian adolescents: social and demographic correlates among a nationally representative sample. PeerJ. 2013;1:e125. 10.7717/peerj.125 24024080 PMC3746957

[31] Smith P, Flay BR, Bell CC, Weissberg RP. The protective influence of parents and peers in violence avoidance among African-American youth. Maternal and Child Health Journal. 2001;5(4):245–52. 10.1023/a:1013080822309 11822526

[32] Springer AE, Sharma S, De Guardado AM, Nava FV, Kelder SH. Perceived parental monitoring and health risk behavior among public secondary school students in El Salvador. The Scientific World Journal. 2006;6:1810–4. 10.1100/tsw.2006.284 17195877 PMC5917349

[33] Lowry R, Cohen LR, Modzeleski W, Kann L, Collins JL, Kolbe LJ. School violence, substance use, and availability of illegal drugs on school property among US high school students. Journal of School Health. 1999;69(9):347–55. 10.1111/j.1746-1561.1999.tb06427.x 10633319

[34] Rudatsikira E, Singh P, Job J, Knutsen S. Variables associated with weapon-carrying among young adolescents in southern California. Journal of Adolescent Health. 2007;40(5):470–3. 10.1016/j.jadohealth.2006.12.011 17448409

[35] Omer M, Shaikh MA, Stiller M, Lowery Wilson M. Physical fighting among school-attending adolescents in El Salvador: an examination of the 2013 Global School-Based Health Survey. International journal of environmental research and public health. 2020;17(4):1248. 10.3390/ijerph17041248 32075210 PMC7068388

[36] World Health Organization, Ministry of Health Royal Government of Bhutan. Report on Bhutan Global School-Based Student Health Survey 2016. 2017.

[37] Andersson N, Paredes-Solís S, Milne D, Omer K, Marokoane N, Laetsang D, et al. Prevalence and risk factors for forced or coerced sex among school-going youth: national cross-sectional studies in 10 southern African countries in 2003 and 2007. BMJ open. 2012;2(2):e000754. 10.1136/bmjopen-2011-000754 22389362 PMC3293138

