# Supplementary material for: Factors associated with physical and sexual violence among school-going adolescents in Nepal: Findings from Global School-based Student Health Survey
Source: PLoS One. 2021 Mar 18;16(3):e0248566. doi: 10.1371/journal.pone.0248566 (PMC7971533; doi:10.1371/journal.pone.0248566)
Supplement: S1 Table — (DOC) [file pone.0248566.s001.doc]

Supplementary file 1

S1 Table: Operational definition of the variables in the study

| **Variable** | **Survey question** | **Coding** |
| --- | --- | --- |
| **Gender** | What is your sex? | 1=Male, 2=female |
| **Age** | How old are you? | 1=12 years or younger, 2=13 years,  3=14 years,  4=15 years,  5=16 years or older |
| **Grade** | In what grade are you? | 1=grade seven  2=grade eight  3=grade nine  4=ten or above |
| **Food insecurity** | During the past 30 days, how often did you go hungry because there was not enough food in your home? | 1=Most of times/always, 0=Never/rarely/sometimes |
| **Physically attacked** | During the past 12 months, how many times were you physically attacked? | 0=0 times,  1=1 or more times |
| **Physical fighting** | During the past 12 months, how many times were you in a physical fight? | 0=0 times,  1=1 or more times |
| **Bullied** | During the past 30 days, how many days were you bullied? | 0=0 times,  1=1 or more times |
| **Felt lonely** | During the past 12 months, how often have you felt lonely? | 1=Most of times/always, 0=Never/rarely/sometimes |
| **Anxiety** | During the past 12 months, how often have you been so worried about something that you could not sleep at night? | 1=Most of times/always, 0=Never/rarely/sometimes |
| **Close Friends** | How many close friends do you have? | 0=0 close friends,  1=1 close friend,  2=2 close friends,  3=3 or more close friends |
| **Current cigarette use** | During the past 30 days, on how many days did you smoke cigarettes? | 0= 0 days  1= 1 or more days |
| **Current cigarette use** | During the past 30 days, on how many days did you smoke cigarettes? | 0=0 times,  1=1 or more times |
| **Current alcohol use** | During the past 30 days, on how many days did you have at least one drink containing alcohol? | 0=0 times,  1=1 or more times |
| **Ever used drug** | How old were you when you first used drugs? | 0= I have never used drugs,  1= any other response |
| **Current marijuana use** | During the past 30 days, how many times have you used marijuana (also called *Gaaja*)? | 0=0 times,  1=1 or more times |
| **Multiple sex partners** | During your life, with how many people have you had sexual intercourse? | 0= I have never had sexual intercourse/1 person  1= 2 or more persons |
| **Truancy** | During the past 30 days, on how many days did you miss classes or school without permission? | 0=0 to 2 times,  1=3 or more times |
| **Parents understand problem** | During the past 30 days, how often did your parents or guardians understand your problems and worries? | 1=Most of times/always, 0=Never/rarely/sometimes |
| **Parental monitoring** | During the past 30 days, how often did your parents or guardians really know what you were doing with your free time? | 1=Most of times/always, 0=Never/rarely/sometimes |
| **Felt unsafe at school** | During the past 30 days, on how many days did you not go to school because you felt you would be unsafe at school or on your way to or from school? | 0=0 days  1= 2 or more days |
| **Corporal punishment** | During the past 12 months, did your teacher ever hit, slap, or physically hurt you on purpose? | 0=No  1=Yes |
