# Supplementary material for: Factors associated with physical and sexual violence among school-going adolescents in Nepal: Findings from Global School-based Student Health Survey
Source: PLoS One. 2021 Mar 18;16(3):e0248566. doi: 10.1371/journal.pone.0248566 (PMC7971533; doi:10.1371/journal.pone.0248566)
Supplement: S2 Table — (DOC) [file pone.0248566.s002.doc]

Supplementary file 2

S2 Table: Results of multiple regression analysis after imputation of missing data

|  | **Physical attack** | | **Physical fight** | | **Sexual violence** | |
| --- | --- | --- | --- | --- | --- | --- |
|  | **AOR (95% CI)** | **p value** | **AOR (95% CI)** | **p value** | **AOR (95% CI)** | **p value** |
| Sex |  |  |  |  |  |  |
| Male | Ref |  | Ref |  | Ref |  |
| Female | 0.78 (0.67, 0.9) | <0.01 | 0.77 (0.61, 0.97) | 0.03 | 1.08 (0.77, 1.5) | 0.66 |
| Age |  |  |  |  |  |  |
| 12 years or under | Ref |  | Ref |  | Ref |  |
| 13 years | 0.77 (0.53, 1.1) | 0.14 | 1.26 (0.83, 1.91) | 0.27 | 0.62 (0.36, 1.05) | 0.07 |
| 14 years | 0.72 (0.51, 1.02) | 0.06 | 1.43 (0.87, 2.36) | 0.16 | 1.08 (0.58, 2) | 0.81 |
| 15 years | 0.64 (0.43, 0.95) | 0.03 | 1.24 (0.75, 2.07) | 0.39 | 1.41 (0.81, 2.47) | 0.22 |
| 16 and above | 0.6 (0.37, 0.95) | 0.03 | 0.99 (0.6, 1.63) | 0.98 | 1.25 (0.71, 2.2) | 0.43 |
| Grade |  |  |  |  |  |  |
| Grade 7 | Ref |  | Ref |  | Ref |  |
| Grade 8 | 0.93 (0.69, 1.25) | 0.60 | 0.87 (0.64, 1.17) | 0.33 | 0.65 (0.39, 1.08) | 0.09 |
| Grade 9 | 0.87 (0.6, 1.27) | 0.46 | 1.09 (0.76, 1.57) | 0.63 | 0.51 (0.32, 0.8) | 0.01 |
| Grade 10 or above | 0.67 (0.46, 0.97) | 0.04 | 1.05 (0.8, 1.39) | 0.71 | 0.32 (0.18, 0.56) | <0.01 |
|  |  |  |  |  |  |  |
| Food Insecurity |  |  |  |  |  |  |
| No | Ref |  | Ref |  | Ref |  |
| Yes | 1.28 (0.9, 1.83) | 0.17 | 0.84 (0.46, 1.54) | 0.56 | 0.95 (0.4, 2.28) | 0.91 |
|  |  |  |  |  |  |  |
| Bullied |  |  |  |  |  |  |
| No | Ref |  | Ref |  | Ref |  |
| Yes | 2.79 (2.25, 3.47) | <0.01 | 3.58 (2.92, 4.39) | <0.01 | 1.25 (0.91, 1.73) | 0.17 |
|  |  |  |  |  |  |  |
| Felt lonely |  |  |  |  |  |  |
| No | Ref |  | Ref |  | Ref |  |
| Yes | 1.29 (0.94, 1.78) | 0.11 | 1.26 (0.79, 2) | 0.33 | 1.14 (0.64, 2.03) | 0.64 |
|  |  |  |  |  |  |  |
| Anxiety |  |  |  |  |  |  |
| No | Ref |  | Ref |  | Ref |  |
| Yes | 1.16 (0.82, 1.63) | 0.39 | 0.96 (0.65, 1.43) | 0.85 | 2.09 (1.03, 4.24) | 0.04 |
|  |  |  |  |  |  |  |
| Close friends |  |  |  |  |  |  |
| No close friend | Ref |  | Ref |  | Ref |  |
| One close friends | 1.55 (0.94, 2.56) | 0.09 | 0.90 (0.53, 1.54) | 0.70 | 0.83 (0.39, 1.76) | 0.61 |
| Two friends | 1.40 (0.86, 2.26) | 0.17 | 0.83 (0.52, 1.33) | 0.43 | 0.98 (0.49, 1.95) | 0.94 |
| Three or more close friends | 1.57 (1.02, 2.42) | 0.04 | 0.79 (0.49, 1.26) | 0.31 | 0.62 (0.31, 1.23) | 0.16 |
|  |  |  |  |  |  |  |
| Smoking |  |  |  |  |  |  |
| No | Ref |  | Ref |  | Ref |  |
| Yes | 1.34 (0.86, 2.09) | 0.18 | 1.38 (0.75, 2.57) | 0.29 | 2.18 (0.84, 5.66) | 0.11 |
|  |  |  |  |  |  |  |
| Drinking |  |  |  |  |  |  |
| No | Ref |  | Ref |  | Ref |  |
| Yes | 1.17 (0.68, 2.01) | 0.57 | 1.22 (0.76, 1.94) | 0.40 | 1.13 (0.59, 2.18) | 0.70 |
|  |  |  |  |  |  |  |
| Marijuana use |  |  |  |  |  |  |
| No | Ref |  | Ref |  | Ref |  |
| Yes | 1.15 (0.6, 2.21) | 0.66 | 1.82 (0.85, 3.89) | 0.12 | 1.12 (0.38, 3.26) | 0.84 |
|  |  |  |  |  |  |  |
| Multiple sexual partner |  |  |  |  |  |  |
| No | Ref |  | Ref |  | Ref |  |
| Yes | 0.87 (0.57, 1.32) | 0.50 | 2.77 (1.34, 5.72) | 0.01 | 2 (1.24, 3.24) | 0.01 |
|  |  |  |  |  |  |  |
| Truancy |  |  |  |  |  |  |
| 0-2 times | Ref |  | Ref |  | Ref |  |
| 3 or more times | 1.31 (0.97, 1.76) | 0.08 | 1.37 (0.87, 2.14) | 0.17 | 1.18 (0.76, 1.83) | 0.46 |
|  |  |  |  |  |  |  |
| Parents understand problem |  |  |  |  |  |  |
| No | Ref |  | Ref |  | Ref |  |
| Yes | 0.92 (0.75, 1.13) | 0.42 | 1.06 (0.91, 1.23) | 0.43 | 0.69 (0.54, 0.87) | <0.01 |
|  |  |  |  |  |  |  |
| Parental monitoring |  |  |  |  |  |  |
| No | Ref |  | Ref |  | Ref |  |
| Yes | 0.82 (0.7, 0.96) | 0.01 | 0.9 (0.73, 1.12) | 0.33 | 0.71 (0.56, 0.91) | 0.01 |
|  |  |  |  |  |  |  |
| Felt unsafe at school |  |  |  |  |  |  |
| No | Ref |  | Ref |  | Ref |  |
| Yes | 1.9 (1.56, 2.32) | <0.01 | 1.15 (0.93, 1.42) | 0.19 | 1.5 (1.09, 2.05) | 0.01 |
